# Inter-laboratory study of an optimised peptide mapping workflow using automated trypsin digestion for monitoring monoclonal antibody product quality attributes

**DOI:** 10.1007/s00216-020-02809-z

**Published:** 2020-07-25

**Authors:** Silvia Millán-Martín, Craig Jakes, Sara Carillo, Tom Buchanan, Marc Guender, Dan Bach Kristensen, Trine Meiborg Sloth, Martin Ørgaard, Ken Cook, Jonathan Bones

**Affiliations:** 1 Characterisation and Comparability Laboratory, National Institute for Bioprocessing Research and Training, Fosters Avenue, Mount Merrion, Blackrock, Co., Dublin, A94 X099 Ireland; 2 School of Chemical and Bioprocess Engineering, University College Dublin, Belfield, Dublin 4, D04 V1W8 Ireland; 3 Thermo Fisher Scientific, Tudor Rd, Runcorn, WA7 1TA UK; 4 Thermo Fisher Scientific, Reinach TechCenter, Neuhofstrasse 11, 4153 Basel, Switzerland; 5 Symphogen, Pederstrupvej 93, 2750 Ballerup, Denmark; 6 Thermo Fisher Scientific, Stafford House, 1 Boundary Park, Hemel Hempstead, HP2 7GE UK

**Keywords:** Inter-laboratory study, Peptide mapping, Monoclonal antibody, Post-translational modifications (PTMs), Trypsin digestion, Method transferability

## Abstract

Peptide mapping analysis is a regulatory expectation to verify the primary structure of a recombinant product sequence and to monitor post-translational modifications (PTMs). Although proteolytic digestion has been used for decades, it remains a labour-intensive procedure that can be challenging to accurately reproduce. Here, we describe a fast and reproducible protocol for protease digestion that is automated using immobilised trypsin on magnetic beads, which has been incorporated into an optimised peptide mapping workflow to show method transferability across laboratories. The complete workflow has the potential for use within a multi-attribute method (MAM) approach in drug development, production and QC laboratories. The sample preparation workflow is simple, ideally suited to inexperienced operators and has been extensively studied to show global applicability and robustness for mAbs by performing sample digestion and LC-MS analysis at four independent sites in Europe. LC-MS/MS along with database searching was used to characterise the protein and determine relevant product quality attributes (PQAs) for further testing. A list of relevant critical quality attributes (CQAs) was then established by creating a peptide workbook containing the specific mass-to-charge (m/z) ratios of the modified and unmodified peptides of the selected CQAs, to be monitored in a subsequent test using LC-MS analysis. Data is provided that shows robust digestion efficiency and low levels of protocol induced PTMs.

**Electronic supplementary material:**

The online version of this article (10.1007/s00216-020-02809-z) contains supplementary material, which is available to authorized users.

**Figure:**
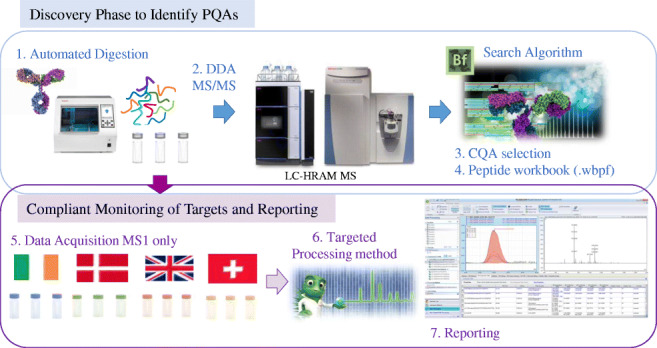
Graphical abstract

## Introduction

Peptide mapping is commonly used in the biopharmaceutical industry to confirm that the desired amino acid sequence of a therapeutic protein has been expressed and to characterise any post-translational modifications (PTM) present [1, 2]. This information supports bioprocess development, lot to lot consistency, biosimilarity assessment [3–6], drug stability in formulation and monitoring the genetic stability of recombinant cell lines [7, 8].

With recent advances in high-resolution accurate mass (HRAM) mass spectrometry instrumentation and semi-automated software platforms, distinguishing between closely related species, and quantitative measurements of these species, using MS has become possible [9]. From the 80 biologic licence applications (BLAs) approved by FDA between 2000 and 2015, 79 BLAs used MS in drug product characterisation [10]. Recently, in 2015, the first paper was published using peptide mapping with HRAM LC-MS as a proposed method to monitor several critical quality attributes using one analytical method in the quality control (QC) laboratory [11]. The described multi-attribute method (MAM) has since then gained considerable popularity and interest throughout the biopharmaceutical community [12]. MAM offers the advantage of measuring multiple protein modifications as product quality attributes (PQAs) during development or critical quality attributes (CQAs) during testing in a single MS run. This specificity is possible due to the bottom-up nature of the approach, where the protein is enzymatically digested to smaller peptides and then analysed by LC-HRAM MS.

Mass spectrometry provides much more detailed information about individual protein modifications than conventional methods [9, 10], offering powerful information such as molecular weight and sequence information (MS/MS) to assist with co-elution challenges, verify sequence coverage and to identify unknown peaks when they appear.

To cope with the increasing numbers of samples and to implement the technique in a QC environment with HRAM LC-MS, sample preparation reproducibility is also required which can be provided by automation through online digestion or robotic systems [13–17].

Digestion procedures vary from laboratory to laboratory and there have been many attempts to optimise the conditions used [1, 2, 17, 18]. The method described in this study overcomes these difficulties by removing many of the steps involved in the traditional digestion procedure. The protein is unfolded using heat denaturation [19, 20] and the digest performed with a heat stable trypsin [21–23]. The elevated temperature used could potentially increase the rate of PTM generation. For example, deamidation during sample preparation is known to increase with time, temperature and pH [11, 24]. The present work shows that using a temperature of 70 °C, deamidation can be negated by lowering the reaction rates for PTM generation by using a reduced pH and increasing the speed of digestion.

This study describes the use of automated digestion as part of a fully optimised, robust, global peptide mapping protocol for monoclonal antibodies, with potential for routine usage in QC laboratories. In preliminary experiments, automated digestion conditions were optimised in terms of digestion time and digestion buffer; results were evaluated by mass analysis of residual intact, undigested protein and by critical PTM evaluation, with a particular focus at lowering sample preparation–induced PTMs, such as deamidation and oxidation. The developed peptide mapping protocol was then applied to investigate data obtained by digestion of the NIST reference antibody standard and LC-MS analysis performed at four laboratories in Europe to assess workflow robustness and ease of method transfer. Additionally, the stability of targeted CQAs present on a mAb mixture under forced degradation conditions was also evaluated.

## Materials and methods

### Chemical and reagents

Trastuzumab and Bevacizumab drug products were kindly provided from a Hospital Pharmacy Unit. The NIST monoclonal antibody (NISTmAb, lot number 14HB-D-002) reference material, RM 8671 (2.4 mg mL^−1^) was purchased from The National Institute of Standards and Technology (NIST). Thermo Scientific™ SMART Digest™ kits, magnetic resin option, were obtained from Thermo Fisher Scientific. LC-MS-grade solvents (0.1% (v/v) formic acid in water, 0.1% (v/v) formic acid in acetonitrile, formic acid, acetonitrile, water) were sourced from Fisher Scientific. Tris(2-carboxyethyl)phosphine hydrochloride (TCEP) and guanidine-HCl were obtained from Pierce. All other reagents were purchased from Sigma-Aldrich.

### Analytical instrumentation

LC-MS analysis was performed using similar but not identical UHPLC systems coupled to Orbitrap-based mass analysers operated with the Thermo Scientific™ Ion Max™ source equipped with the HESI-II-probe (see Electronic Supplementary Material (ESM) Table S1). All data were acquired using Thermo Scientific™ Chromeleon™ Chromatography Data System (CDS) software 7.2.9 (Thermo Fisher Scientific).

### Intact mass analysis

Separations were performed using Thermo Scientific™ MAbPac™ RP 2.1 × 100 mm column (Thermo Fisher Scientific). A binary gradient of 0.1% (v/v) formic acid in water (A) and 0.1% (v/v) formic acid in acetonitrile (B) was used. Gradient conditions were as follows: 5% B to 95% B in 11 min, hold at 95% B until 12 min, 5% B from 12.1 min until 17 min. Column temperature was maintained at 80 °C and a 300 μL min^−1^ flow rate was used. UV coupled online to HRAM MS was used for detection. Full MS acquisition was obtained at a resolution setting of 35,000 (at m/z 200) with a mass range of 1500–4500 m/z and AGC target of 3.0 × 10^6^ with a maximum injection time of 100 ms and 10 microscans. In-source CID was 100 eV. MS tune parameters were as follows: spray voltage was 3.8 kV, sheath gas flow rate was 20 AU, auxiliary gas flow rate was 10 AU, capillary temperature was 300 °C, probe heater temperature was 275 °C and S-Lens RF voltage was set to 100.

### Peptide mapping protocol

Samples were diluted to 2 mg mL^−1^ in water. For each sample digest, sample, digestion buffer (buffer 1, pH 6.5 or buffer 2, pH 7.2) and 5 mM TCEP (final concentration) were added to each lane of a Thermo Scientific™ KingFisher™ deep well 96-well plate as outlined in ESM Table S2, except for one of the laboratories where digestion was performed using the same protocol and magnetic beads manually, employing manual timing and magnetic removal of the beads. Trypsin bead “wash buffer” was prepared by diluting digestion buffer 1:4 (v/v) in water. Bead buffer was neat digestion buffer. Digestion was performed using a Thermo Scientific™ KingFisher™ Duo Prime Purification System with Thermo Scientific™ BindIt™ software (version 4.0). Samples were incubated for 5 to 40 min at 70 °C on medium mixing speed to prevent sedimentation of beads for the digestion time course study and beads were removed at each time point. Following digestion, 100-μL samples were transferred to 300-μL vials and 1 μL of 10% TFA was added (final concentration 0.1% TFA) and immediately analysed by HRAM LC-MS. The tryptic peptides were separated and monitored using a Thermo Scientific™ Acclaim Vanquish™ C18, 2.2 μm, 2.1 × 250 mm (Thermo Fisher Scientific, Cat#074812-V). Analysis was performed using a binary gradient of 0.1% (v/v) formic acid in water (A) and 0.1% (v/v) formic acid in acetonitrile (B). Gradient conditions were as follows: 2% B to 40% B in 105 min, increase to 80% B at 111 min until 115 min, drop to 2% B at 115.5 min until 120 min. The column temperature was maintained at 25 °C throughout and flow rate was sustained at 300 μL min^−1^.

Discovery experiment using data-dependent acquisition (DDA) MS/MS method was performed only from one laboratory and consisted of full positive polarity MS scans at a resolution setting of 70,000. A resolution setting of 140,000 at m/z 200 was used for full MS-targeted monitoring experiments. Mass range was set to 200–2000 m/z and AGC target value of 3.0 × 10^6^ with a maximum injection time of 100 ms and one microscan. In-source CID was set to 0 eV. MS^2^ settings were as follows: a resolution setting of 17,500 (at m/z 200), AGC target value of 1.0 × 10^5^, isolation window set to 2.0 m/z, signal intensity threshold of 1.0 × 10^4^, normalised collision energy set to 28, top 5 precursors selected for fragmentation and dynamic exclusion set to 7 s. MS instrumental tune parameters were set as follows: spray voltage was 3.8 kV, sheath gas flow rate was 25 AU, auxiliary gas flow rate was 10 AU, capillary temperature was 320 °C, probe heater temperature was 150 °C and S-lens RF voltage set to 60.

### Degradation study

ICH stability samples were prepared based on temporal stress (40 °C) for 0, 3 and 6 months. Each sample of each time point was prepared and analysed in true triplicates, i.e. independent digestions, at a concentration of 12 mg/mL per digest. Sample consisted of a mixture of 6 mAbs produced in one of the laboratories involved in the study.

### Data processing

Peptide identification and PTM assessment were performed using Thermo Scientific™ BioPharma Finder™ software version 3.1, according to parameters summarised in ESM Table S3. A target peptide workbook was created containing a list of peptides for the most prominent PTMs including all the detected charge states. The results were selected to include components with up to 1 missed cleavage. Moreover, peptides containing Na^+^/K^+^ adducts were excluded together with non-specific, unknown modifications and gas phase ions as these peptides could be variable and were of too low abundance to significantly change the final values. The target peptide workbook contains information about the selected target peptides that will be used to run a targeted peptide monitoring analysis by exporting the data to a file (.wbpf) compatible with the Chromeleon™ software. ESM Table S4 summarises parameter settings for PTM quantitation using Chromeleon CDS and ESM Table S5 details the MS component table for the studied PTMs. ICIS peak detection was selected as the MS peak detection algorithm.

The analytical instrument method, data processing method and reporting were first created by one laboratory and then transferred as a complete eWorkflow™ procedure to be uploaded on the individual instruments at each site. The results obtained were directly taken from automatic reporting in Chromeleon CDS without any further data manipulation except for the retention time adjustment and peak area integration review. Distribution of experimental parameters as an eWorkflow™ supports the application of identical parameters for data acquisition and data analysis at each site.

## Results and discussion

When performing peptide mapping analysis, the digestion step is by far the most difficult step to reproduce between different operators and laboratories. Without a reproducible, and preferably automated, digestion procedure, the rest of the peptide mapping protocol may be compromised, especially for implementation of the workflow as routine analysis in a QC environment. In particular, peptide mapping protocols may influence correct evaluation of PTM values due to introduction of artificially induced modifications as a result of the numerous steps and excessive sample handling involved. For these reasons, we decided to use temperature-induced denaturation with heat-stable trypsin immobilised on magnetic beads for ease of use and simple automation.

### Intact protein analysis for evaluation of digestion completeness

In preliminary experiments, digestion completeness was investigated for three mAbs (bevacizumab, NISTmAb and trastuzumab) by intact protein analysis using a time course experiment to determine the optimal length of digestion, while minimising the risk of potential experimentally induced modifications. The breakdown and disappearance of the intact mAb to a stable peptide pattern was monitored in the UV trace (data relative to trastuzumab are presented in ESM Fig. S1). The use of magnetic beads allowed the time course to be automated with precise stop points and removal of trypsin at the required times.

Two of the studied mAbs showed the presence of a portion of the protein in some intact form after 60 min (data not shown). RPLC-UV-MS analysis (ESM Fig. S2a) showed a charge envelope under the peak eluting at retention time 7.15–7.50 min for trastuzumab, which upon deconvolution resulted in a mass of approximately 100 kDa (data not shown). While it was not possible to assess the exact nature of this fragment, it was clear that digestion efficacy needed to be boosted. TCEP, a reducing agent that is active at reduced pH [25], was added and significantly improved the digestion efficiency and removed the need for an additional reduction step (ESM Fig. S2b). Loss of trypsin activity was monitored with different TCEP concentrations from 1 to 10 mM. 5 mM TCEP proved to be ideal for enhancing protein digestion by reducing disulphide bonds, also eliminating the need for a reduction step following automated digestion.

The digestion time and the pH of digestion buffer were also investigated to limit the rate of deamidation [24]. Traditionally, trypsin digestion is performed at slightly basic pH of 8 to 8.5; the buffers tested in this study had a pH of 6.5 and 7.2 at room temperature that decreased to pH ~5.9 and 6.5, respectively, when the temperature was elevated to 70 °C. Reduction in pH with elevated temperature is a known phenomenon [26]. The lowest pH value used was that which had no significant effect on the activity of the trypsin used (data not shown). LC conditions are also critical for monitoring PTMs together with column length which would enhance resolution; the optimised gradient conditions are shown in the experimental section and were selected to ensure a good separation of deamidated peptides from the unmodified forms.

### Peptide mapping analysis for digestion time course and PTM study

**Fig. 1:**
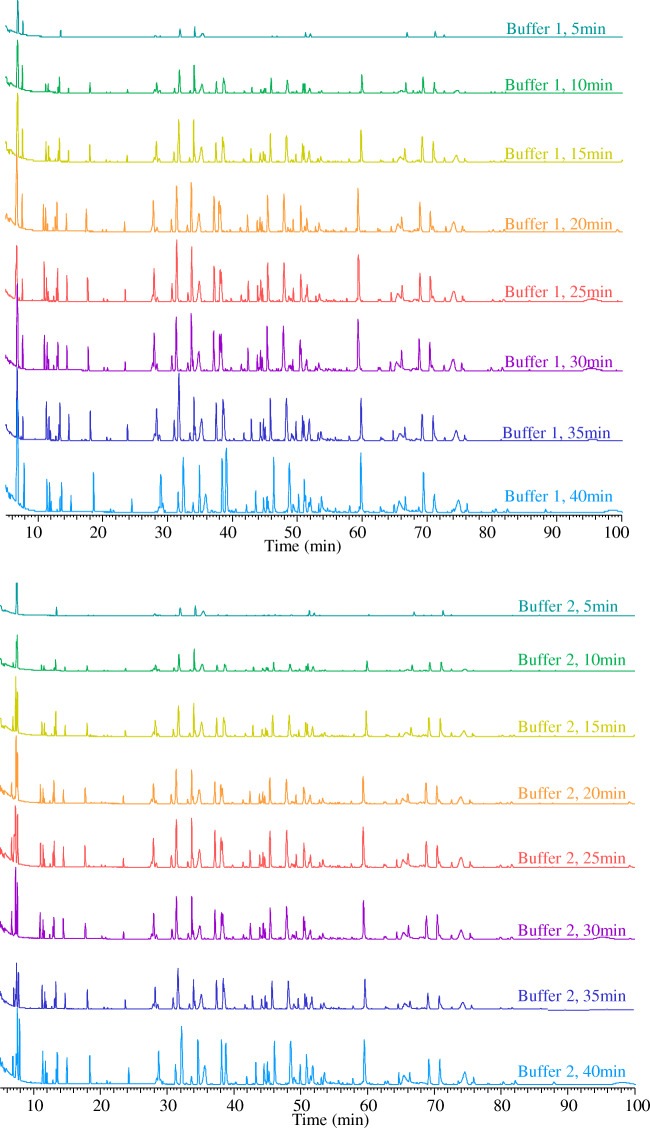
Zoomed view (5–100 min) of stacked base peak chromatograms (BPCs) obtained from peptide mapping experiments of trastuzumab for the digestion time course study using KingFisher™ Duo Prime system for both buffer 1 (pH 6.5) and buffer 2 (pH 7.2) with 5 mM TCEP addition, and without any further reduction or alkylation steps

The time course data indicated that complete digestion was achieved within 30 min for the mAbs used in this study. Figure 1 shows the base peak chromatogram (BPC) obtained from peptide mapping experiments of trastuzumab for the digestion time course study for both buffer 1 (pH 6.5) and buffer 2 (pH 7.2) with 5 mM TCEP addition, and without any further reduction or alkylation steps. One hundred percent sequence coverage was attained for all the time points, even the earlier time points where digestion was observed to be incomplete (ESM, Figs. S3 and S4, and Table S6).

**Fig. 2:**
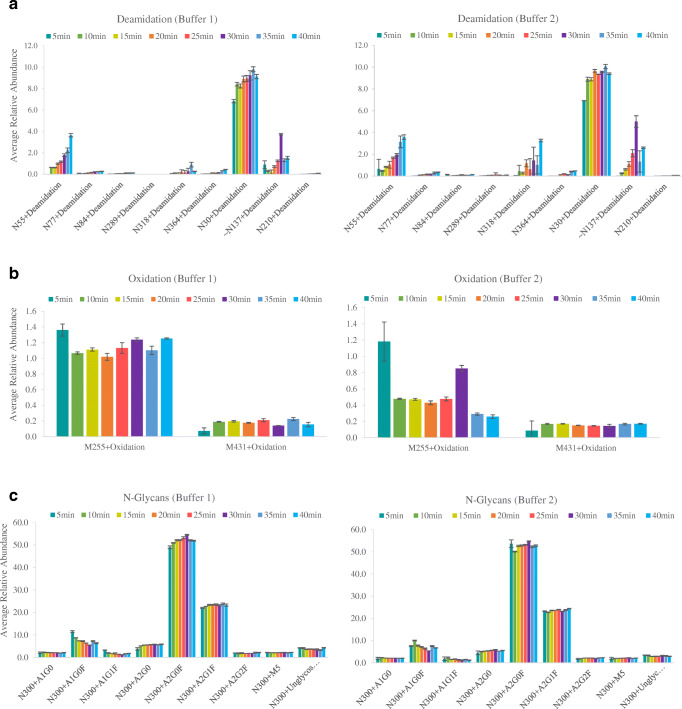
Average relative abundance (*n* = 3) of some identified PTMs: **a** deamidation; **b** oxidation and **c** *N*-glycosylation on the Fc region for trastuzumab time course study using buffer 1 and buffer 2 respectively

**Fig. 3:**
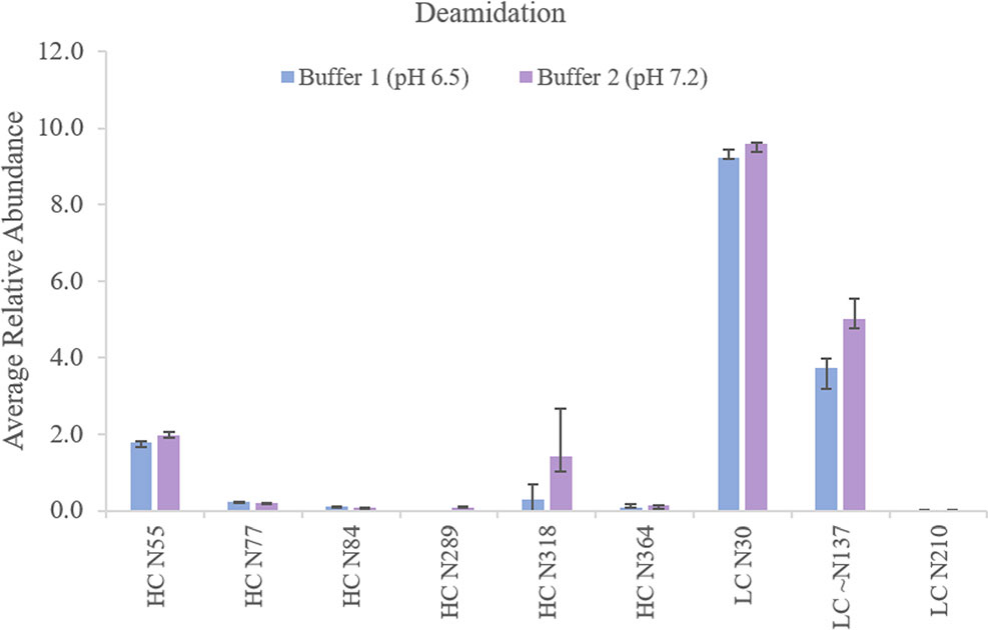
Effect of buffer pH on deamidation levels: average relative abundance (*n* = 3) of deamidated sites for trastuzumab 30-min digest using buffer 2 and buffer 1

Deamidation is the most likely modification to be affected by the high temperature [11, 24]. As such, similar time course experiments were used to monitor the generation of this and other PTMs over time. Nine deamidation events were found, all of which were at a low level (from 0.1 to 2%) apart from HC N55 and LC N30 which were ~4% and 9% respectively, values confirmed by previous reports [27]. Measurement at each time point was conducted in triplicate and produced quantitative results with good standard deviations, even low abundant species (Fig. 2a). There was a small increase in deamidation levels over the digestion time period spanning from 5 to 40 min, which was more noticeable for the HC N55 and LC N30 sites. This indicates good control of the rate of deamidation and would allow longer digestion times if desired. The rate of deamidation will still be dependent on the site of the modification; however, we have not observed any sites which posed a problem during our studies using multiple mAb targets. Comparison between the two buffers did not result in significant differences, except for HC N318 where low pH buffer (pH 6.5) reduced deamidation up to 80% (Fig. 3) although levels were below 2%. Overall, buffer 2 (pH 7.2) showed slightly higher levels of deamidation for HC N55, LC N30 and LC N137 when using a digestion time of 30 min. Buffer 1 reduced digestion-induced deamidation by up to 20 to 80% compared to the levels observed during digestion at pH 7.2 (Fig. 3 and ESM Tables S7 and S8). Levels are also lowered when compared to previously reported data generated using long digestion times in basic buffer [28].

Oxidation is also known to be sensitive to excessive sample handling and suffers from low reproducibility across different analysts and laboratories [29]. During the same time course experiments, the level of oxidation was seen to remain stable for those sites more prone to oxidation (M255 and M431 of the IgG1 HC) when using buffer 1 (Fig. 2b). Using buffer 2, oxidation levels were reduced at the start of the digestion until the levels increased again after 30 min for the M255 site. Those differences have been reported from other laboratories [29, 30] and could be justified by the fact that peptides containing oxidised methionine showed complex elution behaviour and certain variability is due to oxidation processes produced in-sample, in-column and in-source. Oxidation of M431 remained stable along the time course study. In any case, oxidation levels were below 1.5% values and did not increase with digestion time.

Glycosylation was also monitored during the digestion time course. Most abundant glycan structures were monitored closely and the ratio of the relative abundance of each was unchanged and very consistent during the whole time course (Fig. 2c).

The actual values obtained for each modification found during the digestion time course are shown in the ESM as Table S7 and S8 as an average of triplicate sample analysis. The relative standard deviation for peptide peak area response for triplicate digestions is in a very close range for each modification time point, even with very low abundance emerging peptides. Optimised digestion conditions were established for 30 min at 70 °C with buffer 1 according to the observed results for time course study.

### Inter-laboratory peptide mapping study

To ensure the workflow could be potentially utilised in a real QC environment with reliable method transferability, the complete optimised protocol was performed in four different laboratories located in four different countries with different operators. Three of the laboratories used an automated trypsin protocol while only one used the same protocol and magnetic beads manually, employing manual timing and magnetic removal of the beads as detailed before for peptide mapping protocol.

**Fig. 4:**
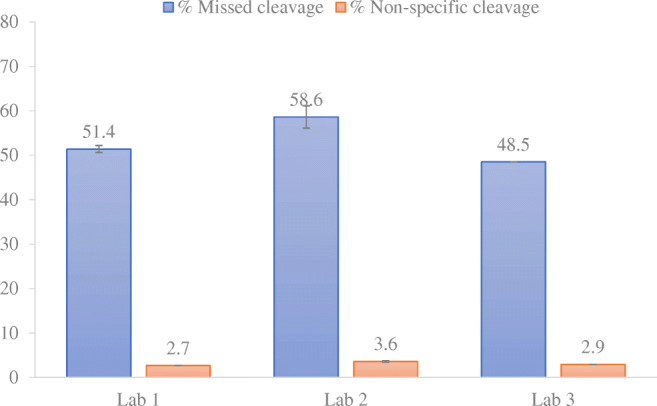
Relative levels of missed cleavage and non-specific cleavage for NISTmAb RM 8671

Missed cleavages and non-specific cleavages were evaluated on NISTmAb MS/MS experiments from 3 sites and calculated as reported by Mouchahoir et al. Missed cleavages values varied from 48.5 to 58.6% between the 3 laboratories with excellent intra-lab RSD values (< 4.3%) and inter-lab RSD < 10%. Relative levels of non-specific generated peptides varied from 2.7 to 3.6% between labs (RSD < 15.4%). Intra-lab precision expressed as RSD value was < 5.3% (Fig. 4).

**Fig. 5:**
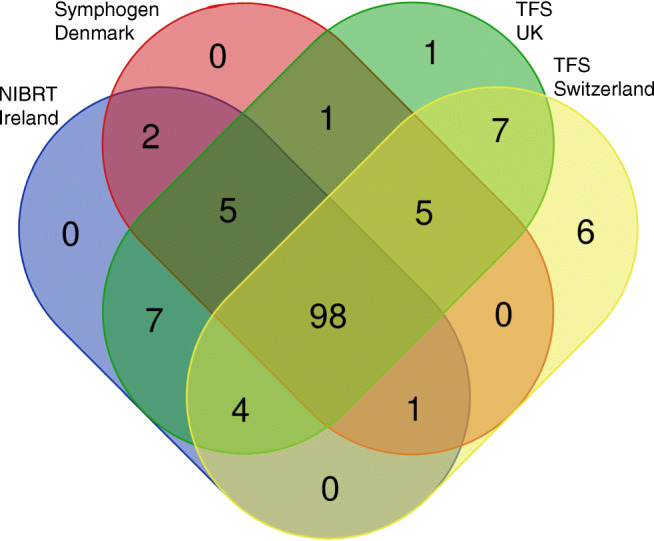
Venn diagram of the peptides identified from automated NISTmAb trypsin digestions performed in four different laboratories. Peptide lists include all the peptides within ± 5 ppm accuracy and including up to one missed cleavage peptides

**Fig. 6:**
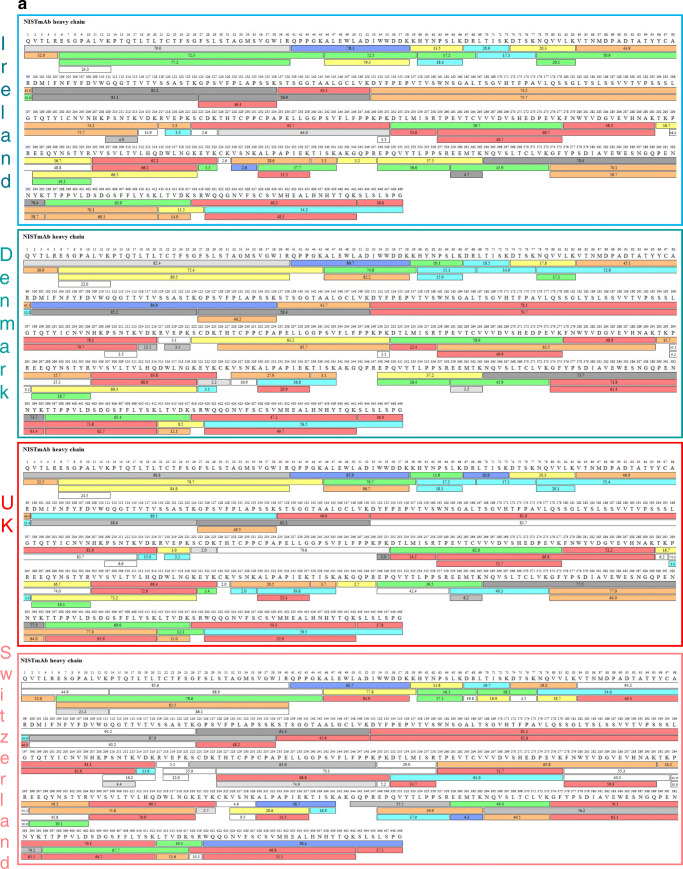

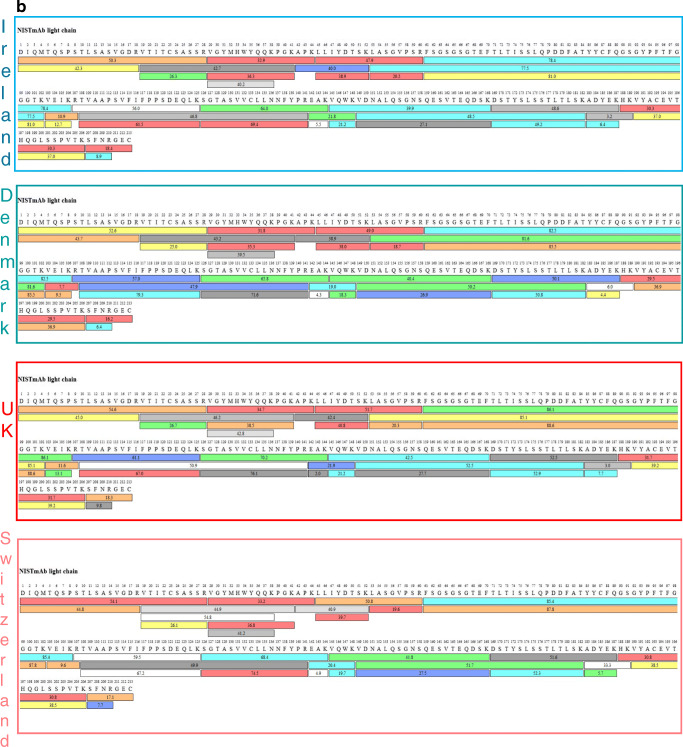
**a** Sequence coverage map of NISTmAb heavy chain obtained from automated trypsin digestions performed in three different laboratories and non-automated digestion performed in Switzerland. The coloured bars show the identified peptides, with the numbers in the bars reflecting the retention time. The different colours indicate the peptide recovery in the MS1 scan: red > 50%, orange > 20% and yellow > 10% represent good recovery. Green, > 5%, light blue > 2% and cyan > 1% represent fair recovery and grey-white scale shows poor recoveries < 1%. **b** Sequence coverage map of NISTmAb light chain obtained from automated trypsin digestions performed in three different laboratories and non-automated digestion performed in Switzerland. The coloured bars show the identified peptides, with the numbers in the bars reflecting the retention time. The different colours indicate the peptide recovery in the MS1 scan: red > 50%, orange > 20% and yellow > 10% represent good recovery. Green, > 5%, light blue > 2% and cyan > 1% represent fair recovery and grey-white scale shows poor recoveries < 1%

The data generated indicate that the digests performed in each laboratory are practically identical including the sites of cleavage and the relative amounts present with a few unique peptides found at very low levels resulting from missed cleavages events, visualised using the Venn diagram [31] in Fig. 5. There are a number of missed cleavages observed with the applied peptide mapping protocol as shown in the sequence coverage maps (Fig. 6a and b); however, several of these are essential to obtain full sequence coverage. A perfect digestion pattern with no cleavage sites missed would generate a high number of small hydrophilic peptides that would not be retained on the C18 column.

To keep the list for the monitoring step more concise, only peptides with no or one missed cleavage were included. Allowing up to only 1 missed cleavage allows these areas of the sequence to be detected without generating an excessively long component list (ESM Table S9). The precision of the digestion ensures that these essential single missed cleavage peptides are always present. The precision of the digests across the labs and locations was excellent. From the total generated peptides, 98 are seen in all four sites with a very minimum number of unique peptides (Fig. 5), even for the non-automated digestion protocols, optimised digestion is robust, easily transferable and no longer a problematic step in the peptide mapping workflow.

**Fig. 7:**
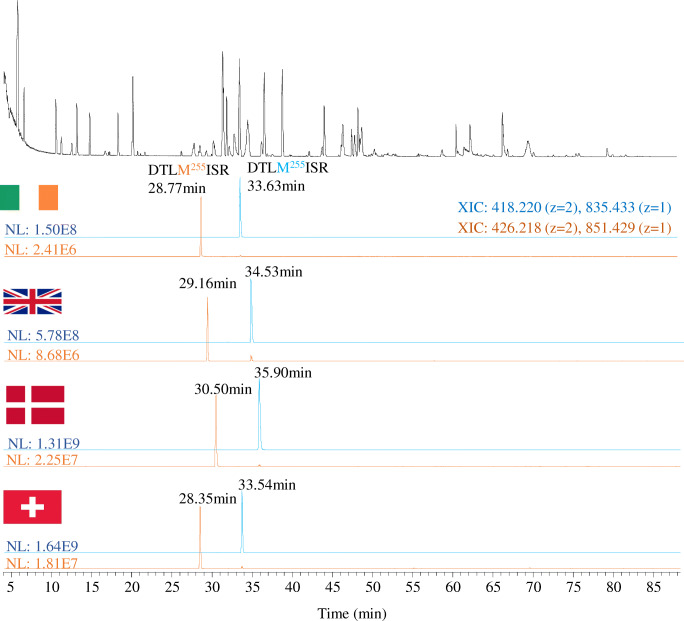
BPC (black trace) and XICs for peptide DTLM255ISR non-modified (blue trace) and oxidised (orange trace) obtained in the four different laboratories

Within the four laboratories, different types of UHPLC pumps were used (ESM Table S1). Due to this and the use of four individual columns, one at each site, the chromatographic separation can be expected to show some degree of variability and thus, the data analysis and reporting method must be flexible to take this into account. As an example, BPC peptide pattern and the extracted ion chromatograms are shown for the peptide DTLMISR with and without the methionine oxidation at M255 in Fig. 7. The retention times for the selected peptides vary by almost 2 min between laboratories. Accurate identification is based on retention time and the accurate mass of the peptide. Due to the high mass accuracy, a wide window can be placed on the peptide retention time to allow for retention time drift in the method. The retention times of the peptides can also be updated manually in the component table as was done for the present study (ESM Table S5).

Relative levels of each PTM were studied from each participating laboratory to compare the quantitative results using triplicate sample analysis. Modifications from the analysis are shown in Table 1 with the values of each PTM monitored from NIST mAb. Reported PTM values showed lower amounts of sample preparation–induced modifications as well as lower variability than other inter-laboratory studies where NIST mAb was also used [29, 30, 32].

**Table 1:** Summary of PTMs identified and quantified for NIST mAb in the four different laboratories using peptide monitoring method and compliant CDS data processing

Modification	Sequence	Relative abundance (*n* = 3)			
		Ireland	Denmark	UK	Switzerland
HC N328+Deam	VSNKALPAPIEK	0.30	0.50	0.33	0.50
	CKVSNK				
	VSNK				
HC N364+Deam	NQVSLTCLVK	0.37	0.20	0.29	0.05
	EEMTKNQVSLTCLVK				
HC~N392/N387+Deam	GFYPSDIAVEWESNGQPENNYK	0.79	0.21	0.24	0.64
HC~N392/N387+Succ	GFYPSDIAVEWESNGQPENNYK	2.06	2.27	2.73	2.60
HC N318+Succ	VVSVLTVLHQDWLNGK	2.05	2.61	2.66	2.25
	VVSVLTVLHQDWLNGKEYK				
HC D283+Succ	FNWYVDGVEVHNAK	2.18	3.40	1.56	3.56
	TPEVTCVVVDVSHEDPEVKFNWYVDGVEVHNAK				
HC M255+Oxid	DTLMISR	1.26	1.29	1.05	0.92
HC K450 Lys loss	SLSLSPGK	87.01	88.49	90.73	88.79
	WQQGNVFSCSVMHEALHNHYTQKSLSLSPGK				
HC Q1+Gln->PyroGlu	QVTLR	99.29	99.31	99.75	99.32
HC N300+M5	EEQYNSTYR	1.71	1.29	1.10	1.22
	TKPREEQYNSTYR				
HC N300+A1G0F	EEQYNSTYR	6.05	5.04	11.26	10.95
	TKPREEQYNSTYR				
HC N300+A2G0F	EEQYNSTYR	41.13	40.14	37.36	37.05
	TKPREEQYNSTYR				
HC N300+A1G1F	EEQYNSTYR	4.06	3.68	5.92	6.13
	TKPREEQYNSTYR				
HC N300+A2G1F	EEQYNSTYR	36.11	38.65	33.86	34.89
	TKPREEQYNSTYR				
HC N300+A2G2F	EEQYNSTYR	6.92	8.49	7.02	7.31
	TKPREEQYNSTYR				
HC N300+A2Ga1G1F	EEQYNSTYR	0.91	1.42	1.09	1.21
	TKPREEQYNSTYR				
HC N300+unglycos	EEQYNSTYR	3.06	1.29	2.39	1.25
	TKPREEQYNSTYR				

Analysis of variance (ANOVA) was used to evaluate intra- and inter-laboratory precision [33, 34]. It is important to point out that the relative levels of the studied CQAs will impact the precision values, observing the highest variability for relative abundances < 2% of deamidation levels (ESM Table S10). Intra-laboratory precision expressed as the relative standard deviation (RSD) was overall < 10% for most PTMs, except for deamidation of N364 and ~N392/N387 whose values were between 42 and 20%, respectively. Inter-laboratory precision demonstrated the highest variability for deamidation of N364, relative abundances varied from 0.05 to 0.37%, and deamidation of ~N392/N387 where relative abundances varied from 0.29 to 0.79%. Excellent precision was observed for oxidation, Lys loss, pyroglutamate formation and the *N*-glycosylation levels (ESM Table S10).

**Fig. 8:**
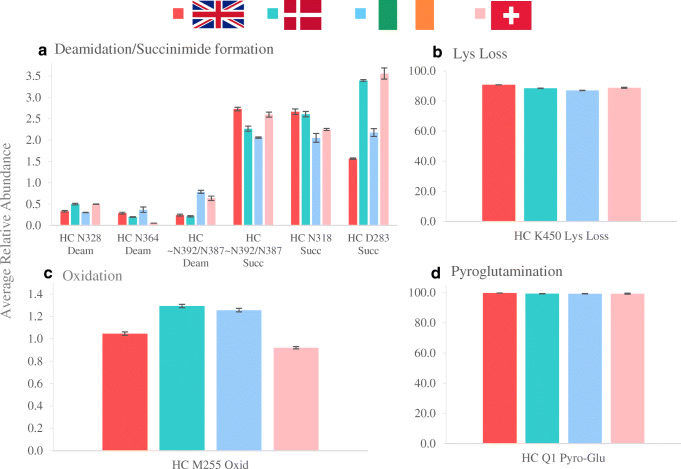
Comparison of the qualitative results for PTM values from 4 different laboratories for the analysis of NIST mAb digestions: deamidation/succinimide formation (**a**); Lys loss (**b**); oxidation (**c**) and pyroglutamination (**d**) of NIST mAb

The levels of deamidation and succinimide formation are all below 2% and 3.6%, respectively, and show comparable values at all four sites. The formation of succinimide and the conversion to isoaspartic acid and aspartic acid is an equilibrium reaction that will depend on environmental conditions, so some variation in the numbers can be expected. However, in this study, the comparative results are still close (Fig. 8a, Table 1).

The lysine loss measurement from the C-terminus was comparable between laboratories from 87 to 90% (Fig. 8b, Table 1). Inter-laboratory precision was excellent giving high confidence in the results (RSD < 3%). The M255 oxidation (Fig. 8c, Table 1) is very low yet only varied between 0.8 and 1.2% between laboratories with a much tighter tolerance within the triplicate injections of the same laboratory (RSD < 3.5%). N-terminal glutamine (Gln) to pyroglutamate (PyroGlu) was also monitored in this study and was shown to be at completion in all four laboratories (Fig. 8d, Table 1).

**Fig. 9:**
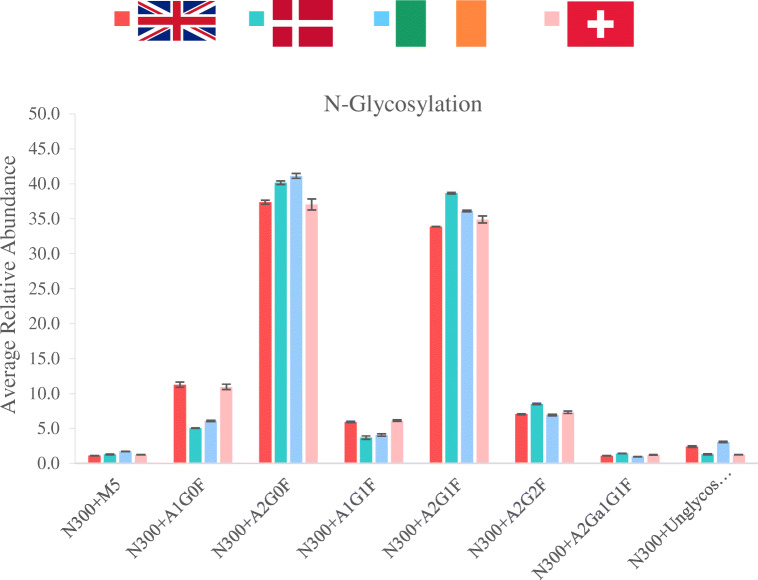
*N*-Glycosylation of NIST mAb over four laboratories. The combined area counts from EEQYN^300^STYR and the single missed cleavage product TKPREEQYN^300^STYR were used in the final evaluated result shown

As expected, glycosylation profile proved to be very stable and consistent across sites, as confirmed by the laboratory comparison results shown in Fig. 9. All four laboratories returned similar *N*-glycosylation data with minimal variability for most of the most abundant glycoforms (intra-lab precision < 10% and inter-lab precision from 9.9 to 67.2%). Reported N-glycans were over 1% of relative levels, and no sialylated glycan structure is shown as they were detected at low levels (< 1%), which is in accordance with reported values for NISTmAb [32].

Sequence coverage map comparison from each individual instrument is shown in Fig. 6a and b for heavy and light chains respectively. Full sequence coverage is achieved for NISTmAb heavy and light chains, except for two of the laboratories where > 98.5% was achieved for HC. In those instances, the missing peptide corresponded to 1:A341-R347 (AKGQPR) which can also be detected in peptides containing 2 missed cleavage events (TISKAKGQPR and AKGQPREPQVYTLPPSR).

### Inter-laboratory stability study of degraded mAb mixture

The developed peptide mapping protocol was then applied to investigate the stability of targeted CQAs present on a mAb mixture under forced degradation conditions. Two different laboratories (site A and site B) received the same samples which had been left to degrade at 40 °C for several months. Chosen modifications were tracked in each sample using the same experimental procedures as previously described. Table 2 shows the comparative results from the two individual laboratories for the targeted attributes. Values in parenthesis show the RSD of the triplicate analysis for each of the studied CQAs, showing excellent precision for each laboratory with overall RSD < 10%.

**Table 2:** Summary of PTMs identified and quantified for the stability study of mAb mixture in two different laboratories using peptide monitoring and compliant CDS data processing

Sequence	Protein	Avg rel. abundance 40 °C_0 months (*n* = 3)		Avg rel. abundance 40 °C_3 months (*n* = 3)		Avg rel. abundance 40 °C_6 months (*n* = 3)	
		Site A	Site B	Site A	Site B	Site A	Site B
KGNYGNYGK	mAb4_HC N101+deamidation	6.02 (1.81)	4.81 (5.57)	18.81 (3.33)	16.04 (5.12)	21.61 (1.37)	24.23 (3.35)
ASQDINNYLNWYQQKPGK	mAb6_LC~N30+deamidation	1.27 (7.54)	0.46 (8.08)	28.08 (1.14)	25.52 (0.67)	44.68 (1.40)	43.65 (0.16)
GFYPSDIAVEWESNGQPENNYK	mAb1_HC~N389+deamidation	1.66 (6.33)	0.19 (7.41)	2.54 (7.91)	0.44 (5.78)	3.28 (8.33)	0.69 (3.70)
GFYPSDIAVEWESNGQPENNYK	mAb1_HC~N384+NH3 loss	1.70 (5.48)	0.33 (3.99)	1.99 (3.84)	0.34 (4.26)	1.98 (2.30)	0.30 (5.57)
VVSVLTVLHQDWLNGK	mAb1_HC N315+deamidation	0.96 (0.52)	0.65 (6.29)	1.19 (4.25)	0.70 (3.99)	1.48 (1.88)	0.61 (3.79)
VVSVLTVLHQDWLNGK	mAb1_HC N315+NH3 loss	3.89 (4.69)	2.67 (4.28)	4.31 (3.43)	2.68 (4.09)	4.59 (6.29)	2.47 (2.98)
ASQDVDTAVAWYQQKPGK	mAb2_LC~D30+isomerisation		3.42 (3.96)	2.33 (5.63)	47.75 (3.63)	40.66 (1.08)	45.92 (1.43)
ASQDVDTAVAWYQQKPGK	mAb2_LC~D30+H2O loss	0.16 (5.79)	0.13 (9.86)	2.14 (3.50)	2.52 (2.81)	2.03 (4.38)	2.83 (0.48)
FNWYVDGVEVHNAK	mAb1_HC D280+isomerisation	0.73 (6.71)	0.57 (3.32)	6.06 (2.11)	4.71 (3.71)	9.59 (2.86)	7.74 (2.55)
FNWYVDGVEVHNAK	mAb1_HC D280+H2O loss	0.06 (3.63)	0.22 (2.97)	0.17 (4.71)	0.37 (1.11)	0.18 (2.00)	0.35 (3.12)
DTLMISR	mAb1_HC M252+oxidation	3.40 (2.25)	2.81 (5.04)	5.93 (3.19)	4.94 (2.19)	5.82 (2.20)	5.59 (1.84)
DIQMTQSPSSLSASVGDR	mAb3_LC M4+oxidation	1.79 (7.23)	0.06 (19.61)	2.46 (6.82)	0.09 (5.89)	1.77 (3.75)	0.09 (8.60)
LLMYISR	mAb3_LC M48+oxidation	0.45 (7.93)	–	0.59 (6.12)	–	0.582 (1.70)	–
WQQGNVFSCSVMHEALHNHYTQK	mAb1_HC M428+oxidation	1.87 (6.04)	1.16 (6.90)	2.35 (4.69)	1.91 (3.68)	2.38 (12.20)	2.27 (3.21)
LLIYWASTR	mAb2_LC W50+oxidation	0.18 (2.80	0.24 (5.37)	0.55 (0.38)	0.70 (8.58)	0.87 (7.23)	1.01 (4.93)
DVVMTQSPLSLPVTLGQPASISCR	mAb1_LC M4+oxidation	1.85 (2.51)	0.56 (4.53)	5.04 (1.96)	3.26 (1.56)	5.20 (9.02)	3.63 (1.15)
ASGYTFTSYWMQWVR	mAb2_HC W33+oxolactone	0.20 (3.94)	0.35 (4.06)	3.97 (6.33)	6.89 (20.13)	6.18 (0.64)	12.17 (5.54)

**Fig. 10:**
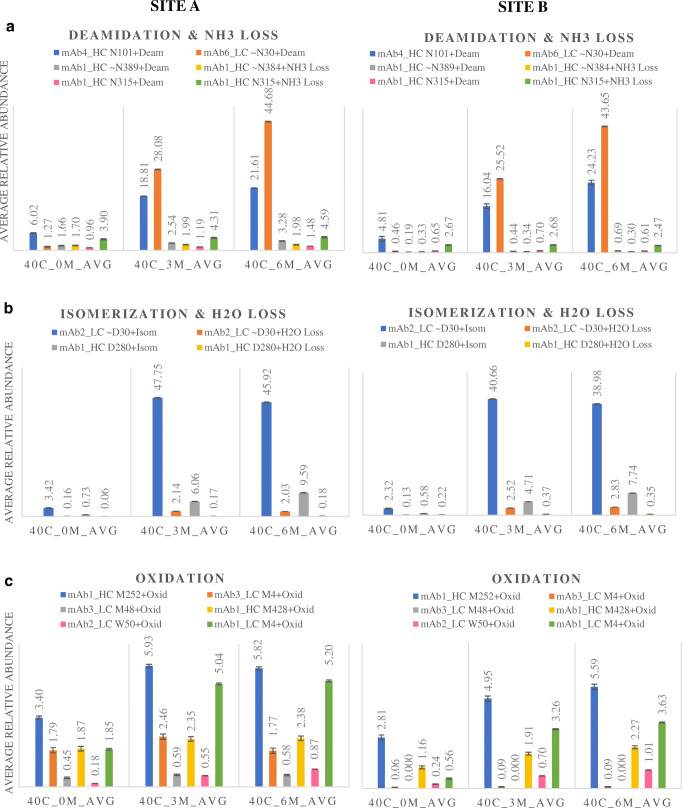
Degradation study using the complete compliant peptide mapping workflow. Comparison of the qualitative results for targeted PTM values from 2 different laboratories for the analysis of thermal stressed samples. Deamidation/NH3 loss (**a**); isomerisation/H2O loss (**b**); oxidation (**c**)

Deamidation levels rose throughout the incubation period at 40 °C (Fig. 10a) for those asparagine (N) residues more prone to deamidation, i.e. the LC N30 and HC N101 sites. Aspartic acid isomerisation also increased for LC D30 after 3 months and then maintained stable at 6 months (Fig. 10b). Oxidation levels also tended to slightly increase over time and maintained stable at 6 months (Fig. 10c), especially for HC M252 and LC M4 sites for the studied mAb mixture.

## Conclusions

We demonstrated a complete peptide monitoring workflow including the digestion, transferred seamlessly across different laboratories. While the aim of this work is to prove workflow robustness and accuracy, this could also be considered as a preliminary study to the implementation of a MAM approach in a QC lab. The results suggest that it is indeed possible to deliver a method to QC environments in various geographies that brings the benefit of HRAM MS data to the characterisation of therapeutic monoclonal antibodies.

A simple digestion protocol has been developed and tested which is easily automated using magnetic beads enabling operators to generate a predictable, precise and robust digestion each time irrespective of the user or location. Each stage of the method has been optimised for ease of use as well as functionality, including the use of compliant automated control and data processing which would otherwise be prone to individual interpretation. Instrument control and reporting was secured in a Chromeleon eWorkflow which was transferred to each laboratory. The automated digestion method described here has been tested using a multitude of mAb samples to ensure global applicability with fast and easy implementation and provides very high precision of digestion. The digest occurs in a controlled and precise manner and allows robust tracking of PTMs. The target peptides included in the component table were selected to ensure correct reproducible results of all the attributes measured while keeping the component list as simple as possible. The method was transferred between different laboratories to show excellent precision of the digestion and corresponding results. The chosen monitored PTMs were shown to be easily tracked in a forced degradation study with the same inter-laboratory precision.

## Electronic supplementary material

ESM 1 (PDF 1498 kb)
