# Supplementary material for: Inter-laboratory study of an optimised peptide mapping workflow using automated trypsin digestion for monitoring monoclonal antibody product quality attributes
Source: Anal Bioanal Chem. 2020 Jul 25;412(25):6833–48. doi: 10.1007/s00216-020-02809-z (PMC7496030; doi:10.1007/s00216-020-02809-z)
Supplement: Supplementary file 1 — (PDF 1498 kb) [file 216_2020_2809_MOESM1_ESM.pdf]

**Analytical and Bioanalytical Chemistry**

**Electronic Supplementary Material**

# Interlaboratory study of an optimised peptide mapping workflow using automated trypsin digestion for monitoring monoclonal antibody product quality attributes

Silvia Millán-Martín, Craig Jakes, Sara Carillo, Tom Buchanan, Marc Guender,
Dan Bach Kristensen, Trine Meiborg Sloth, Martin Ørgaard, Ken Cook, Jonathan Bones

Additional files available under 10.1007/s00216-020-02809-z

**Table S1** .......................... Analytical Instrumentation

**Table S2** .......................... 96-well plate scheme for trypsin digestion

**Table S3** .......................... BPF software parameter settings for peptide mapping data analysis

**Table S4** .......................... CDS software parameter settings for peptide mapping data processing and PTMs quantitation.

**Table S5** .......................... MS Component table of CDS software for the studied PTMs (see separate .xlsx file)

**Figure S1**......................... UV Chromatogram traces (280nm) for trastuzumab RP-UV-MS Intact analysis during digestion time course study using buffer 1

**Figure S2**......................... Base Peak Chromatograms (BPC) for trastuzumab RP-UV-MS Intact Analysis using 5mM TCEP as reducing agent (a), and without TCEP addition (b)

**Figure S3**......................... Sequence coverage map of trastuzumab obtained using automated tryptic digestion with low pH buffer 1 after 5min.

**Figure S4**......................... Sequence coverage map of trastuzumab obtained using automated tryptic digestion with SMART buffer 2 after 5min

**Table S6** .................... Sequence coverage for the time course of digestion for trastuzumab

**Table S7** .......................... Summary of PTMs identified and quantified for trastuzumab after digestion with trypsin beads, low pH buffer and 5mM TCEP addition on the KingFisher Duo Prime system

**Table S8** .......................... Summary of PTMs identified and quantified for trastuzumab after digestion with trypsin beads, SMART buffer and 5mM TCEP addition on the KingFisher Duo Prime system

**Table S9** .......................... Component list from BPF3.1 software for sequence coverage and Venn diagram (see separate .xlsx file)

**Table S10** ........................ Analysis of Variance (ANOVA) to measure intra and inter-lab precision for NISTmAb complete compliant peptide mapping workflow

**Table S1** Analytical Instrumentation used for the 4 Laboratories across Europe

| | Site A (Ireland) | Site B (Denmark) | Site C (UK) | Site D (Switzerland) |
|---|---|---|---|---|
| UHPLC | Vanquish™ Flex Binary | Vanquish™ Duo Ternary | Vanquish™ Horizon Binary | Vanquish™ Flex Binary |
| HRMS | Q Exactive™ Plus | Q Exactive™ Plus | Q Exactive™ Plus | Q Exactive™ Plus |
| SMART Digest | Magnetic Beads KingFisher™ Duo | Magnetic Beads KingFisher™ Duo | Magnetic Beads KingFisher™ Duo | Manual Magnetic beads |

**Table S2** 96-well plate scheme for trypsin digestion

| Lane | Content | Volume (µL) |
|---|---|---|
| **A** | Low pH buffer | 150 |
| | Sample (100µg) | 10 |
| | TCEP (final concentration 5mM) | 2 |
| | Water | 38 |
| **B** | Tip Comb | |
| **C** | Empty | |
| **D** | Magnetic SMART™ Beads | 15 |
| | Bead Buffer (Low pH buffer) | 100 |
| **E** | Bead Wash Buffer (Bead Buffer 1:4 (v/v)) | 200 |
| **F** | Waste Lane (Water) | 250 |

**Table S3** BPF software parameter settings for peptide mapping data analysis

| **Component Detection** | **Setting** |
|---|---|
| Absolute MS Signal Threshold | 1.0 x $10^4$ counts |
| Typical chromatographic peak width | 0.3 |
| Relative MS signal threshold (% base peak) | 1 |
| Relative Analog threshold (% of highest peak) | 1 |
| Width of Gaussian filter (represented as 1/n of chromatographic peak width) | 3 |
| Minimum valley to be considered as two chromatographic peaks | 80.0% |
| Minimum MS peak width (Da) | 1.2 |
| Maximum MS peak width (Da) | 4.2 |
| Mass tolerance (ppm for high-res or Da for low-res) | 4.00 |
| Maximum retention time shift (min) | 1.00 |
| Maximum mass (Da) | 30,000 |
| Mass Centroiding Cutoff (% from base) | 15 |
| **Identification** | **Setting** |
| Maximum peptide mass | 7,000 |
| Mass Accuracy | 5ppm |
| Minimum Confidence | 0.8 |
| Maximum Number of Modifications for a Peptide | 1 |
| Unspecified Modification | -58 to +162 Da |
| N-Glycosylation | CHO |
| Protease Specificity | High |
| **Static Modifications** | **Setting** |
| Side Chain | - |
| **Variable Modifications** | **Setting** |
| N Terminal | Gln - > Pyro Glu |
| C Terminal | Loss of lysine |
| Side Chain | Deamidation (N)<br>Deamidation(Q)<br>Glycation (K)<br>Oxidation (MW)<br>AsnAsu (N)<br>AspAsu (D) |

**Table S4** Chromeleon$^{TM}$ Data System 7.2.9 parameter settings for peptide mapping data processing and PTMs quantitation

| MS Detection | Setting |
|---|---|
| Extracted Ion Chromatogram | MS Default Detection Settings |
| Detection Algorithm | ICIS |
| Area noise factor | 5 |
| Peak noise factor | 10 |
| Baseline window | 40 |
| Noise method | INCOS |
| Min peak width | 3 |
| Multiplet resolution | 10 |
| Area tail extension | 5 |
| Area scan window | 0 |
| **MS Component table** | **Setting** |
| Supplementary Table S5 (.xlsx file) | .wbpf workbook from BPF3.1 |
| **MS Settings** | **MS Chromatogram Settings** |
| Mass Precision | 5 decimal places |
| Mass Tolerance (manually defined) | 8.0 ppm |
| Smoothing | none |
| **Peptide Table** | **Setting** |
| Imported from BPF3.1 peptide workbook | - |
| **Composite Scoring** | **Setting** |
| Pass score if at least | 2 criteria passed |
| Fail score if less than | 1 criteria passed |
| MS Criteria | General MS |
| Isotopic dot product | ≥ 0.9000 |
| Mass accuracy | ≤ 5.00 ppm |
| Peal apex alignment | ≤ 0.50 min |

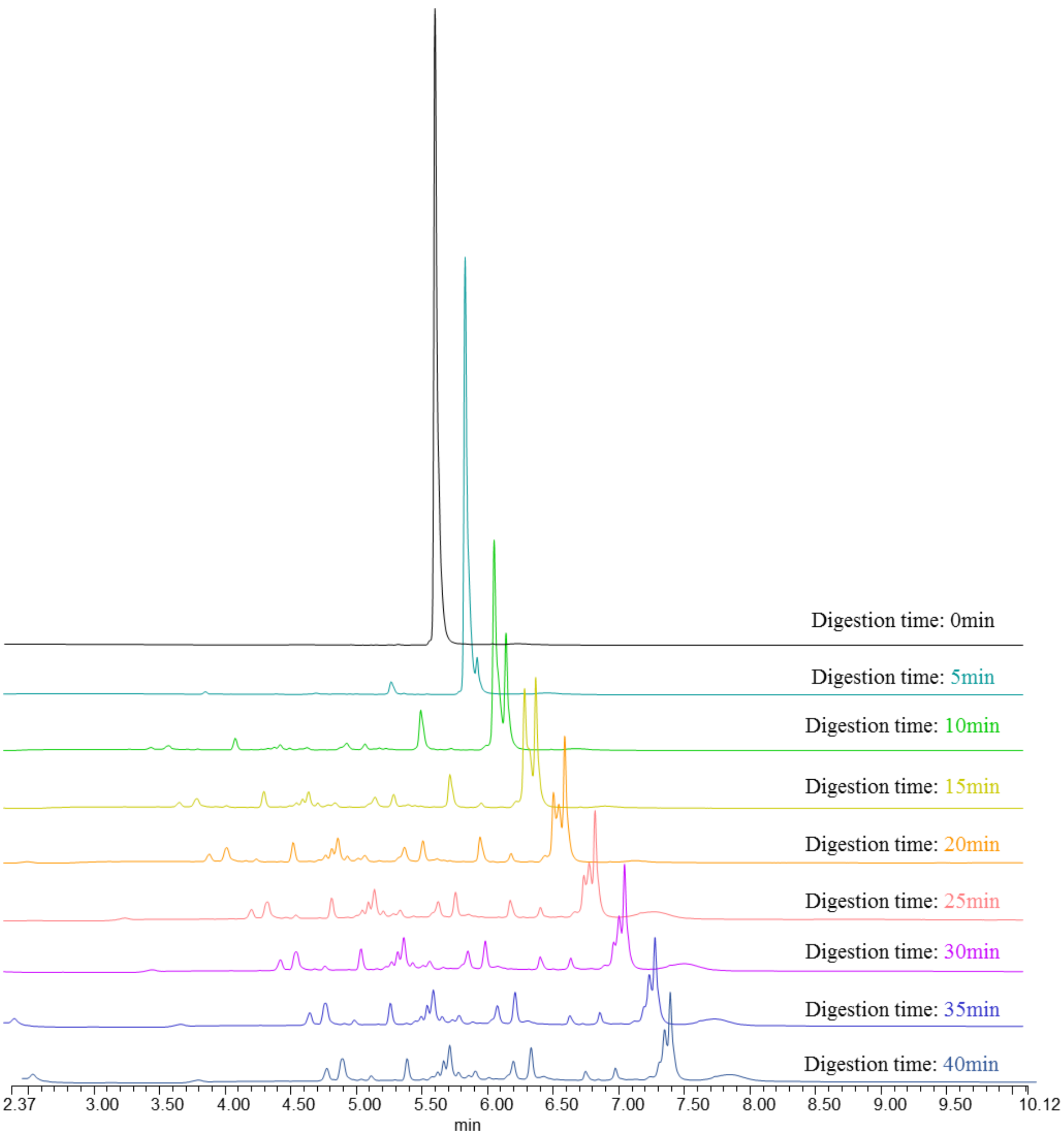


**Fig. S1** UV Chromatogram traces (280nm) for trastuzumab RP-UV-MS Intact analysis during digestion time course study using buffer 1

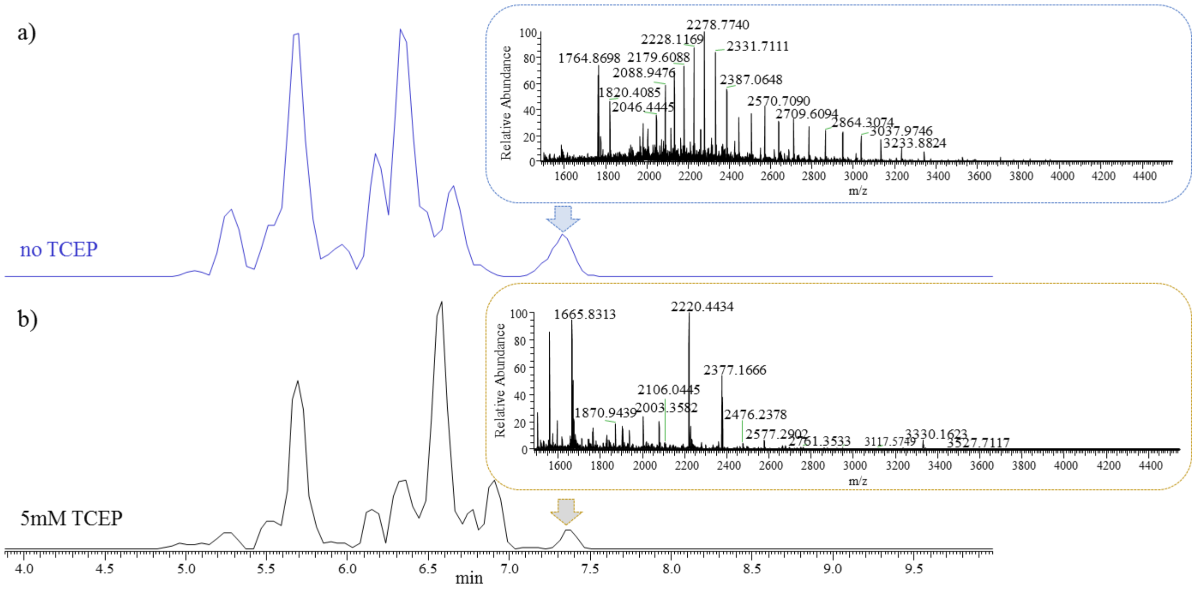


**Fig. S2** Base Peak Chromatograms (BPC) for trastuzumab RP-UV-MS Intact Analysis using 5mM TCEP as reducing agent (a), and without TCEP addition (b). Digestion was performed for 35min and buffer 2

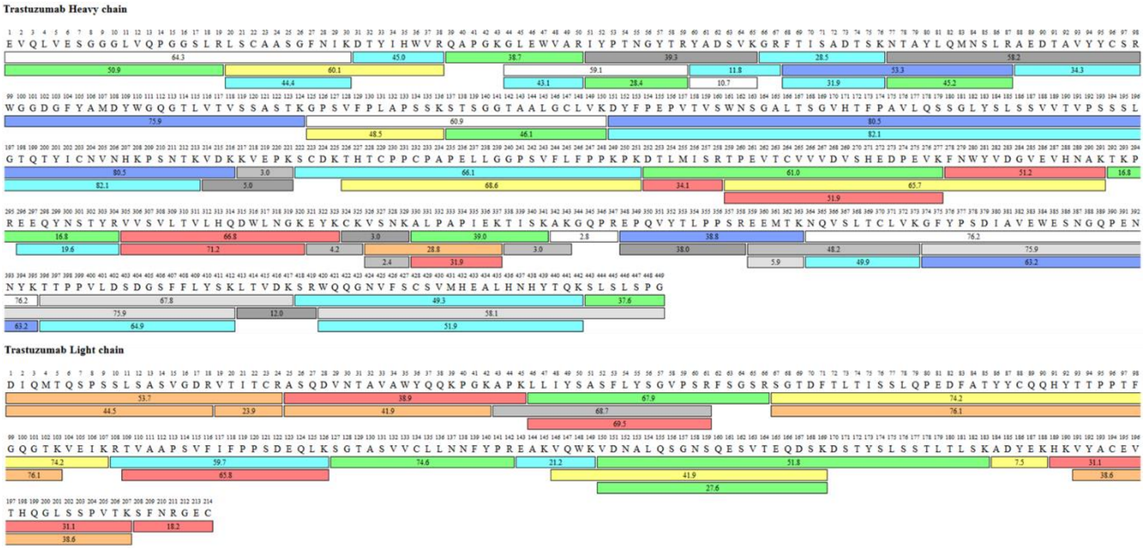

**Fig. S3** Sequence coverage map of trastuzumab heavy (upper panel) and light (lower panel) chains, obtained using automated tryptic digestion with buffer 1 after 5 minutes. The coloured bars show the identified peptides, with the numbers in the bars reflecting the retention time. The different colors indicate the peptide recovery in the MS1 scan: red >50%, orange >20% and yellow >10% represent good recovery. Green, >5%, light blue >2% and cyan >1% represent fair recovery and grey-white scale shows poor recovered peptides

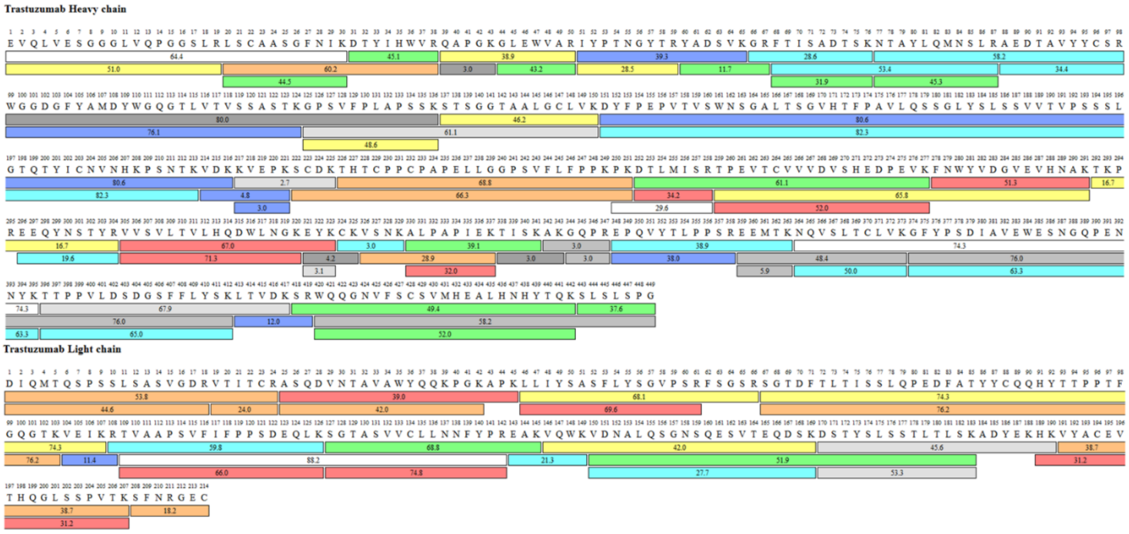

**Fig. S4** Sequence coverage map of trastuzumab heavy (upper panel) and light (lower panel) chains, obtained using automated tryptic digestion with buffer 2 after 5 minutes. The coloured bars show the identified peptides, with the numbers in the bars reflecting the retention time. The different colors indicate the peptide recovery in the MS1 scan: red >50%, orange >20% and yellow >10% represent good recovery. Green, >5%, light blue >2% and cyan >1% represent fair recovery and grey-white scale shows poor recovered peptides

**Table S6** Sequence coverage for the time course of digestion for trastuzumab

| Proteins | | Avg Number of MS Peaks (n=3) | Average Sequence Coverage (n=3) |
|---|---|---|---|
| **Buffer 1, 5min** | HC | 353 | 100% |
| | LC | 103 | 100% |
| **Buffer 1, 10min** | HC | 449 | 100% |
| | LC | 134 | 100% |
| **Buffer 1, 15min** | HC | 510 | 100% |
| | LC | 152 | 100% |
| **Buffer 1, 20min** | HC | 578 | 100% |
| | LC | 167 | 100% |
| **Buffer 1, 25min** | HC | 570 | 100% |
| | LC | 161 | 100% |
| **Buffer 1, 30min** | HC | 553 | 100% |
| | LC | 158 | 100% |
| **Buffer 1, 35min** | HC | 536 | 100% |
| | LC | 147 | 100% |
| **Buffer 1, 40min** | HC | 594 | 100% |
| | LC | 180 | 100% |
| **Buffer 2, 5min** | HC | 353 | 100% |
| | LC | 95 | 100% |
| **Buffer 2, 10min** | HC | 548 | 100% |
| | LC | 156 | 100% |
| **Buffer 2, 15min** | HC | 593 | 100% |
| | LC | 169 | 100% |
| **Buffer 2, 20min** | HC | 617 | 100% |
| | LC | 172 | 100% |
| **Buffer 2, 25min** | HC | 621 | 100% |
| | LC | 165 | 100% |
| **Buffer 2, 30min** | HC | 599 | 100% |
| | LC | 155 | 100% |
| **Buffer 2, 35min** | HC | 555 | 100% |
| | LC | 149 | 100% |
| **Buffer 2, 40min** | HC | 649 | 100% |
| | LC | 172 | 100% |

**Table S7** Summary of PTMs identified and quantified for trastuzumab after digestion with trypsin beads, buffer 1 and 5mM TCEP addition on the KingFisher™ Duo Prime system

| Modification | 5min (n=3) | 10min (n=3) | 15min (n=3) | 20min (n=3) | 25min (n=3) | 30min (n=3) | 35min (n=3) | 40min (n=3) |
|---|---|---|---|---|---|---|---|---|
| HC N55+Deamidation | — | 0.63 | 0.61 | 0.98 | 1.17 | 1.78 | 2.23 | 3.64 |
| HC N77+Deamidation | 0.07 | 0.04 | 0.06 | 0.12 | 0.16 | 0.24 | 0.25 | 0.26 |
| HC N84+Deamidation | 0.02 | 0.02 | 0.04 | 0.07 | 0.07 | 0.12 | 0.12 | 0.12 |
| HC N289+Deamidation | 0.00 | 0.00 | 0.00 | 0.00 | 0.00 | 0.00 | 0.00 | 0.00 |
| HC N318+Deamidation | 0.05 | 0.11 | 0.05 | 0.22 | 0.16 | 0.28 | 0.90 | 0.26 |
| HC N364+Deamidation | 0.01 | 0.04 | 0.02 | 0.12 | 0.09 | 0.09 | 0.33 | 0.44 |
| LC N30+Deamidation | 6.82 | 8.41 | 8.24 | 8.89 | 8.94 | 9.23 | 9.80 | 9.12 |
| LC ~N137+Deamidation | 0.88 | 0.30 | 0.21 | 0.72 | 1.26 | 3.73 | 1.30 | 1.53 |
| LC N210+Deamidation | 0.00 | 0.00 | 0.00 | 0.00 | 0.01 | 0.02 | 0.02 | 0.07 |
| HC M255+Oxidation | 1.36 | 1.06 | 1.11 | 1.02 | 1.13 | 1.24 | 1.10 | 1.25 |
| HC M431+Oxidation | 0.07 | 0.19 | 0.19 | 0.18 | 0.21 | 0.14 | 0.22 | 0.15 |
| HC G449+Lys | 1.08 | 1.44 | 1.60 | 1.62 | 1.66 | 1.60 | 1.83 | 1.69 |
| HC N55+AsnAsu | 1.00 | 1.21 | 1.45 | 2.14 | 2.36 | 2.93 | 3.39 | 3.60 |
| HC N318+AsnAsu | 0.48 | 1.05 | 1.74 | 2.77 | 3.38 | 3.93 | 3.41 | 3.90 |
| LC N30+AsnAsu | 0.19 | 1.48 | 1.53 | 1.08 | 0.87 | 0.50 | 0.50 | 0.31 |
| LC ~N138+AsnAsu | 9.67 | 1.49 | 1.71 | 3.06 | 3.87 | 7.43 | 2.04 | 1.92 |

**Table S7 (continued)** Summary of PTMs identified and quantified for trastuzumab after digestion with trypsin beads, buffer 1 and 5mM TCEP addition on the KingFisher™ Duo Prime system

| Modification | 5min (n=3) | 10min (n=3) | 15min (n=3) | 20min (n=3) | 25min (n=3) | 30min (n=3) | 35min (n=3) | 40min (n=3) |
|---|---|---|---|---|---|---|---|---|
| HC ~D102+AspAsu | 0.21 | 0.48 | 0.73 | 0.92 | 1.10 | 1.52 | 1.27 | 1.47 |
| HC ~D283+AspAsu | 0.53 | 1.49 | 2.57 | 2.68 | 2.57 | 4.02 | 3.64 | 4.54 |
| HC ~D404+AspAsu | 0.28 | 0.03 | 0.01 | 1.43 | 1.64 | 1.56 | 1.29 | 1.033 |
| HC D224+isomerization | 69.11 | 55.64 | 70.37 | 52.95 | 66.25 | 66.19 | 75.22 | 84.46 |
| HC N300+A1G0 | 2.07 | 2.35 | 2.16 | 2.15 | 2.10 | 2.10 | 2.00 | 2.16 |
| HC N300+A1G0F | 11.43 | 8.76 | 7.43 | 7.28 | 6.40 | 5.48 | 7.27 | 6.32 |
| HC N300+A1G1F | 3.26 | 1.99 | 1.75 | 1.76 | 1.45 | 1.19 | 1.64 | 1.78 |
| HC N300+A2G0 | 3.78 | 5.16 | 5.46 | 5.56 | 5.69 | 5.86 | 5.62 | 5.82 |
| HC N300+A2G0F | 48.99 | 50.91 | 52.15 | 52.18 | 53.23 | 54.56 | 52.07 | 51.93 |
| HC N300+A2G1F | 22.04 | 22.69 | 23.41 | 23.44 | 23.50 | 23.19 | 23.86 | 23.27 |
| HC N300+A2G2F | 2.00 | 1.84 | 1.91 | 1.75 | 1.80 | 1.79 | 2.16 | 2.17 |
| HC N300+M5 | 2.17 | 2.11 | 2.08 | 2.15 | 2.16 | 2.29 | 2.05 | 2.26 |
| HC N300+Unglycosylated | 4.26 | 4.18 | 3.63 | 3.73 | 3.65 | 3.53 | 3.32 | 4.28 |

**Table S8** Summary of PTMs identified and quantified for trastuzumab after digestion with trypsin beads, buffer 2 and 5mM TCEP addition on the KingFisher™ Duo Prime system

| Modification | 5min (n=3) | 10min (n=3) | 15min (n=3) | 20min (n=3) | 25min (n=3) | 30min (n=3) | 35min (n=3) | 40min (n=3) |
|---|---|---|---|---|---|---|---|---|
| HC N55+Deamidation | 0.64 | 0.48 | 0.85 | 1.04 | 1.69 | 1.95 | 3.14 | 3.58 |
| HC N77+Deamidation | 0.01 | 0.02 | 0.08 | 0.13 | 0.15 | 0.19 | 0.29 | 0.36 |
| HC N84+Deamidation | 0.14 | 0.02 | 0.05 | 0.07 | 0.09 | 0.09 | 0.08 | 0.15 |
| HC N289+Deamidation | 0.01 | 0.02 | 0.04 | 0.05 | 0.14 | 0.075 | 0.04 | 0.09 |
| HC N318+Deamidation | 0.05 | 0.43 | 0.28 | 1.17 | 0.62 | 1.43 | 1.023 | 3.26 |
| HC N364+Deamidation | 0.00 | 0.01 | 0.01 | 0.13 | 0.21 | 0.13 | 0.42 | 0.47 |
| LC N30+Deamidation | 6.92 | 8.90 | 8.88 | 9.65 | 9.34 | 9.58 | 10.06 | 9.40 |
| LC ~N137+Deamidation | 0.00 | 0.27 | 0.62 | 1.11 | 2.10 | 5.00 | 1.33 | 2.60 |
| LC N210+Deamidation | 0.00 | 0.00 | 0.00 | 0.01 | 0.02 | 0.03 | 0.04 | 0.07 |
| HC M255+Oxidation | 1.18 | 0.48 | 0.47 | 0.43 | 0.48 | 0.85 | 0.29 | 0.26 |
| HC M431+Oxidation | 0.09 | 0.17 | 0.17 | 0.15 | 0.15 | 0.14 | 0.17 | 0.17 |
| HC G449+Lys | 0.57 | 1.44 | 1.53 | 1.63 | 1.63 | 1.56 | 1.81 | 1.76 |
| HC N55+AsnAsu | 1.30 | 1.22 | 1.89 | 2.29 | 2.73 | 3.24 | 3.57 | 3.52 |
| HC N318+AsnAsu | 0.51 | 1.52 | 3.03 | 6.31 | 5.49 | 9.19 | 3.90 | 4.44 |
| LC N30+AsnAsu | 0.31 | 1.32 | 0.89 | 0.73 | 0.48 | 0.39 | 0.27 | 0.21 |
| LC ~N137+AsnAsu | 0.00 | 0.00 | 0.02 | 0.02 | 0.00 | 0.00 | 0.00 | 0.00 |

**Table S8 (continued)** Summary of PTMs identified and quantified for trastuzumab after digestion with trypsin beads, buffer 2 and 5mM TCEP addition on the KingFisher™ Duo Prime system.

| Modification | 5min (n=3) | 10min (n=3) | 15min (n=3) | 20min (n=3) | 25min (n=3) | 30min (n=3) | 35min (n=3) | 40min (n=3) |
|---|---|---|---|---|---|---|---|---|
| HC ~D102+AspAsu | 0.37 | 0.16 | 0.80 | 0.81 | 0.96 | 1.38 | 1.14 | 1.11 |
| HC ~D283+AspAsu | 0.90 | 0.46 | 4.02 | 6.45 | 5.28 | 6.92 | 3.57 | 4.54 |
| HC ~D404+AspAsu | 0.03 | 1.32 | 2.02 | 2.21 | 1.13 | 1.84 | 0.68 | 1.90 |
| HC D224+isomerization | 0.03 | 52.43 | 45.51 | 45.68 | 64.12 | 54.92 | 49.51 | 47.36 |
| HC N300+A1G0 | 1.98 | 2.35 | 2.15 | 2.07 | 2.10 | 2.10 | 2.11 | 2.04 |
| HC N300+A1G0F | 7.54 | 10.03 | 7.75 | 7.12 | 6.38 | 5.43 | 7.56 | 6.73 |
| HC N300+A1G1F | 1.85 | 2.35 | 1.51 | 1.72 | 1.34 | 1.16 | 1.52 | 1.17 |
| HC N300+A2G0 | 4.48 | 5.03 | 5.29 | 5.42 | 5.64 | 5.93 | 5.29 | 5.51 |
| HC N300+A2G0F | 53.65 | 50.11 | 52.68 | 52.89 | 53.19 | 54.71 | 52.33 | 52.80 |
| HC N300+A2G1F | 23.28 | 22.74 | 23.59 | 23.64 | 23.99 | 23.17 | 23.82 | 24.43 |
| HC N300+A2G2F | 1.77 | 2.05 | 2.15 | 2.18 | 2.17 | 1.98 | 2.22 | 2.27 |
| HC N300+M5 | 1.99 | 2.01 | 2.03 | 2.06 | 2.16 | 2.26 | 2.02 | 2.14 |
| HC N300+Unglycosylated | 3.45 | 3.33 | 2.84 | 2.89 | 3.02 | 3.26 | 3.12 | 2.90 |

**Table S10** Analysis of Variance (ANOVA) to measure intra and inter-lab precision for NISTmAb complete compliant peptide mapping workflow

HC N328+Deam

| *Source of Variation* | *SS* | *df* | *MS* | *F* | *P-value* | *F crit* |
|---|---|---|---|---|---|---|
| Between Groups | 0.1001 | 3 | 0.0334 | 153.9872 | 2.05E-07 | 4.0662 |
| Within Groups | 0.0017 | 8 | 0.0002 | | | |
| Intra-Lab precision (RSD) | **10.22** | | | | | |
| Inter-Lab precision (RSD) | **44.43** | | | | | |

HC N364+Deam

| *Source of Variation* | *SS* | *df* | *MS* | *F* | *P-value* | *F crit* |
|---|---|---|---|---|---|---|
| Between Groups | 0.1659 | 3 | 0.0553 | 49.1556 | 1.69E-05 | 4.0662 |
| Within Groups | 0.0090 | 8 | 0.0011 | | | |
| Intra-Lab precision (RSD) | **42.16** | | | | | |
| Inter-Lab precision (RSD) | **101.64** | | | | | |

HC ~N392/N387+ Deam

| *Source of Variation* | *SS* | *df* | *MS* | *F* | *P-value* | *F crit* |
|---|---|---|---|---|---|---|
| Between Groups | 0.7405 | 3 | 0.2468 | 219.4049 | 5.09E-08 | 4.0662 |
| Within Groups | 0.0090 | 8 | 0.0011 | | | |
| Intra-Lab precision (RSD) | **20.22** | | | | | |
| Inter-Lab precision (RSD) | **105.25** | | | | | |

HC D283+Succ

| *Source of Variation* | *SS* | *df* | *MS* | *F* | *P-value* | *F crit* |
|---|---|---|---|---|---|---|
| Between Groups | 8.3645 | 3 | 2.7882 | 420.8549 | 3.86E-09 | 4.0662 |
| Within Groups | 0.0530 | 8 | 0.0066 | | | |
| Intra-Lab precision (RSD) | **8.61** | | | | | |
| Inter-Lab precision (RSD) | **62.24** | | | | | |

HC N318+Succ

| *Source of Variation* | *SS* | *df* | *MS* | *F* | *P-value* | *F crit* |
|---|---|---|---|---|---|---|
| Between Groups | 0.7734 | 3 | 0.2578 | 52.6100 | 1.31E-05 | 4.0662 |
| Within Groups | 0.0392 | 8 | 0.0049 | | | |
| Intra-Lab precision (RSD) | **8.28** | | | | | |
| Inter-Lab precision (RSD) | **20.68** | | | | | |

HC ~N392/N387+Succ

| *Source of Variation* | *SS* | *df* | *MS* | *F* | *P-value* | *F crit* |
|---|---|---|---|---|---|---|
| Between Groups | 0.8415 | 3 | 0.2805 | 125.5970 | 4.56E-07 | 4.0662 |
| Within Groups | 0.0179 | 8 | 0.0022 | | | |

| | |
|---|---|
| Intra-Lab precision (RSD) | **5.54** |
| Inter-Lab precision (RSD) | **21.73** |

**Table S10 (continued)** Analysis of Variance (ANOVA) to measure intra and inter-lab precision for NISTmAb complete compliant peptide mapping workflow

HC M255+Oxid

| *Source of Variation* | *SS* | *df* | *MS* | *F* | *P-value* | *F crit* |
|---|---|---|---|---|---|---|
| Between Groups | 0.2813 | 3 | 0.0938 | 468.8194 | 2.51E-09 | 4.0662 |
| Within Groups | 0.0016 | 8 | 0.0002 | | | |
| Intra-Lab precision (RSD) | **3.54** | | | | | |
| Inter-Lab precision (RSD) | **27.04** | | | | | |

HC K450 Lys Loss

| *Source of Variation* | *SS* | *df* | *MS* | *F* | *P-value* | *F crit* |
|---|---|---|---|---|---|---|
| Between Groups | 21.0218 | 3 | 7.0073 | 225.5554 | 4.57E-08 | 4.0662 |
| Within Groups | 0.2485 | 8 | 0.0311 | | | |
| Intra-Lab precision (RSD) | **0.56** | | | | | |
| Inter-Lab precision (RSD) | **2.96** | | | | | |

HC Q1+Gln->Pyro-Glu

| *Source of Variation* | *SS* | *df* | *MS* | *F* | *P-value* | *F crit* |
|---|---|---|---|---|---|---|
| Between Groups | 0.4365 | 3 | 0.1455 | 831.3651 | 2.57E-10 | 4.0662 |
| Within Groups | 0.0014 | 8 | 0.0002 | | | |
| Intra-Lab precision (RSD) | **0.04** | | | | | |
| Inter-Lab precision (RSD) | **0.38** | | | | | |

HC N300+M5

| *Source of Variation* | *SS* | *df* | *MS* | *F* | *P-value* | *F crit* |
|---|---|---|---|---|---|---|
| Between Groups | 0.6293 | 3 | 0.2098 | 143.0152 | 2.74E-07 | 4.0662 |
| Within Groups | 0.0117 | 8 | 0.0015 | | | |
| Intra-Lab precision (RSD) | **8.14** | | | | | |
| Inter-Lab precision (RSD) | **34.11** | | | | | |

**Table S10 (continued)** Analysis of Variance (ANOVA) to measure intra and inter-lab precision for NISTmAb complete compliant peptide mapping workflow

HC N300+A1G0F

| *Source of Variation* | *SS* | *df* | *MS* | *F* | *P-value* | *F crit* |
|---|---|---|---|---|---|---|
| Between Groups | 94.4941 | 3 | 31.4980 | 438.9969 | 3.26E-09 | 4.0662 |
| Within Groups | 0.5740 | 8 | 0.0718 | | | |
| Intra-Lab precision (RSD) | **9.10** | | | | | |
| Inter-Lab precision (RSD) | **67.20** | | | | | |

HC N300+A2G0F

| *Source of Variation* | *SS* | *df* | *MS* | *F* | *P-value* | *F crit* |
|---|---|---|---|---|---|---|
| Between Groups | 36.8955 | 3 | 12.2985 | 55.2535 | 1.09E-05 | 4.0662 |
| Within Groups | 1.7807 | 8 | 0.2226 | | | |
| Intra-Lab precision (RSD) | **3.43** | | | | | |
| Inter-Lab precision (RSD) | **8.79** | | | | | |

HC N300+A1G1F

| *Source of Variation* | *SS* | *df* | *MS* | *F* | *P-value* | *F crit* |
|---|---|---|---|---|---|---|
| Between Groups | 14.1954 | 3 | 4.7318 | 183.6998 | 1.03E-07 | 4.0662 |
| Within Groups | 0.2061 | 8 | 0.0258 | | | |
| Intra-Lab precision (RSD) | **9.18** | | | | | |
| Inter-Lab precision (RSD) | **43.65** | | | | | |

HC N300+A2G1F

| *Source of Variation* | *SS* | *df* | *MS* | *F* | *P-value* | *F crit* |
|---|---|---|---|---|---|---|
| Between Groups | 38.3946 | 3 | 12.7982 | 196.0907 | 7.93E-08 | 4.0662 |
| Within Groups | 0.5221 | 8 | 0.0653 | | | |
| Intra-Lab precision (RSD) | **2.01** | | | | | |
| Inter-Lab precision (RSD) | **9.90** | | | | | |

HC N300+A2G2F

| *Source of Variation* | *SS* | *df* | *MS* | *F* | *P-value* | *F crit* |
|---|---|---|---|---|---|---|
| Between Groups | 4.7004 | 3 | 1.5668 | 104.6852 | 9.28E-07 | 4.0662 |
| Within Groups | 0.1197 | 8 | 0.0150 | | | |
| Intra-Lab precision (RSD) | **4.65** | | | | | |
| Inter-Lab precision (RSD) | **16.62** | | | | | |

HC N300+ A2Ga1G1F

| *Source of Variation* | *SS* | *df* | *MS* | *F* | *P-value* | *F crit* |
|---|---|---|---|---|---|---|
| Between Groups | 0.3505 | 3 | 0.1168 | 175.2333 | 1.23E-07 | 4.0662 |
| Within Groups | 0.0053 | 8 | 0.0007 | | | |
| Intra-Lab precision (RSD) | **6.24** | | | | | |
| Inter-Lab precision (RSD) | **28.99** | | | | | |
